# Clinical Outcomes and Healthcare Costs of CART Versus Paracentesis for Malignant Ascites: A Nationwide Retrospective Cohort Study in Japan

**DOI:** 10.1002/cam4.71491

**Published:** 2025-12-26

**Authors:** Yuki Hashimoto, Norihiko Inoue, Shinobu Imai

**Affiliations:** ^1^ Department of Clinical Data Management and Research, Clinical Research Center National Hospital Organization Headquarters Tokyo Japan; ^2^ Department of Pharmacoepidemiology Showa University Graduate School of Pharmacy Tokyo Japan

**Keywords:** CART, in‐hospital mortality, malignant ascites, paracentesis, resource allocation

## Abstract

**Background:**

Paracentesis temporarily relieves malignant ascites but causes hypoalbuminemia. Cell‐free and concentrated ascites reinfusion therapy (CART) reinfuses autologous proteins to prevent hypoalbuminemia and has been increasingly used in Japan. However, CART has not been widely adopted outside of Japan, and its benefit remains unclear. We evaluated the clinical outcomes and healthcare costs of CART compared with paracentesis in metastatic cancer.

**Methods:**

This retrospective cohort study included hospitalized patients with metastatic solid cancer receiving CART or paracentesis across Japan (April 2016–March 2023). Baseline characteristics were balanced using overlap propensity‐score weighting. Primary outcomes were in‐hospital mortality, functional disability, and 30‐day unplanned readmission. Secondary outcomes were length of stay (LOS), albumin administration or re‐drainage rates, and costs. Mortality risk was assessed using a modified Poisson regression. The composite primary outcomes were assessed using a win‐ratio approach.

**Results:**

Among 1159 patients (CART: 457, paracentesis: 702) from 51 hospitals, the CART group had lower mortality than the paracentesis group (28.6% vs. 36.7%; risk ratio: 0.78, 95% confidence intervals [95% CI]: 0.64–0.94). The win‐ratio analysis also favored the CART group over the paracentesis group (win ratio: 1.34, 95% CI: 1.09–1.64). Additionally, CART was associated with lower mortality and better composite outcomes than paracentesis, particularly among males, patients with serum albumin ≤ 2.5 g/dL, and those with non‐gastrointestinal cancer. Despite higher procedural costs, CART was associated with shorter median LOS (14.1 vs. 19.0 days), lower albumin administration (11.6% vs. 17.3%) and re‐drainage (32.7% vs. 52.7%) rates, and lower total median costs (4490.9 [interquartile range: 2042.3–7054.5] vs. 5084.1 [interquartile range: 3054.7–8659.7] USD) than paracentesis.

**Conclusions:**

CART was associated with improved clinical outcomes and healthcare costs over paracentesis among hospitalized patients with metastatic cancer, particularly in males, patients with serum albumin ≤ 2.5 g/dL, and those with non‐gastrointestinal cancer. These findings may support clinical decision‐making and resource allocation.

## Graphical abstract

Cell‐free and concentrated ascites reinfusion therapy had lower mortality and in‐hospital costs than paracentesis in metastatic cancer, particularly in males, patients with serum albumin ≤ 2.5 g/dL, and those with non‐gastrointestinal cancer.

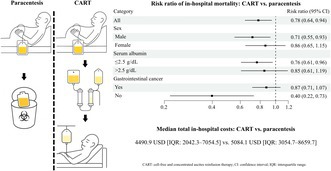

## 1 Introduction

Malignant ascites is a common complication in advanced cancer, particularly in gastrointestinal and gynecological cancers [1, 2, 3]. Patients with malignant ascites experience symptoms, including abdominal distension, dyspnea, appetite loss, and renal dysfunction. These symptoms not only impair quality of life (QOL) but also may result in lost opportunities for chemotherapy [4].

Ascites drainage by paracentesis is performed to alleviate symptoms and improve QOL among patients with refractory ascites [5]; however, it often requires repeated procedures [6]. This frequent drainage can lead to substantial protein loss and hypoalbuminemia. Albumin supplementation is recommended after large‐volume paracentesis, but the repeated consumption of albumin, a limited resource derived solely from blood donations, raises both ethical and economic concerns [7]. Without adequate albumin replacement, patients may experience further deterioration in their nutritional and functional condition, creating a clinical dilemma [8]. An alternative approach, peritoneovenous shunting, may prevent hypoalbuminemia, but could lead to postoperative complications such as disseminated intravascular coagulation [9].

**FIGURE 1:**
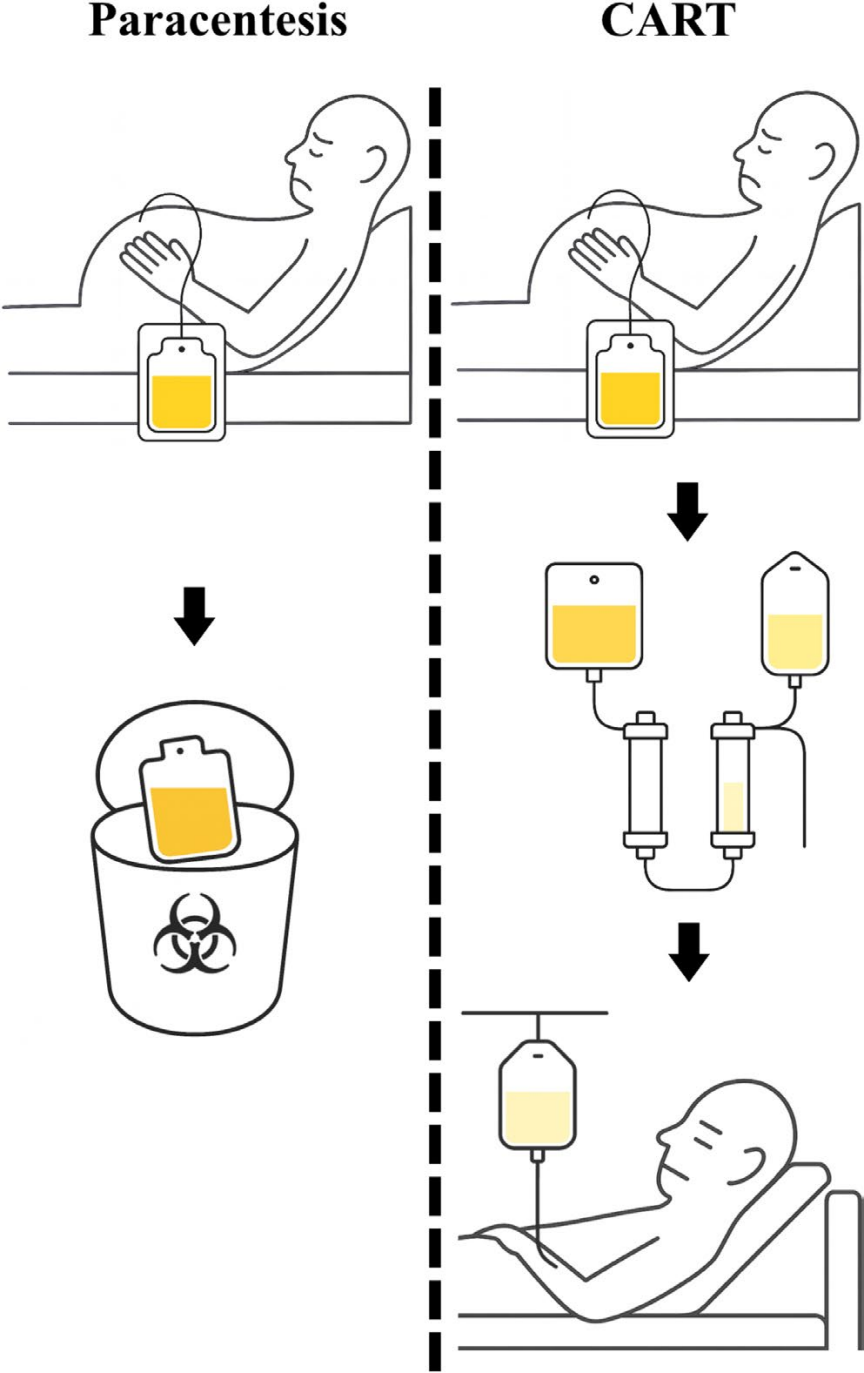
Schematic diagram of CART and paracentesis. CART, cell‐free and concentrated ascites reinfusion therapy.

To counter the protein loss and safety concerns, cell‐free and concentrated ascites reinfusion therapy (CART), an extracorporeal filtration system, was developed in Japan [4, 10]. CART employs a dual‐membrane system that removes malignant cells and bacteria by filtration while concentrating the remaining proteins by eliminating excess water, enabling the reinfusion of autologous concentrated proteins back to the patient (Figure 1) [11]. This reinfusion system may help to maintain nutrition status, lead to early improvements in QOL, create opportunities for further treatments such as chemotherapy, and finally enhance survival [12]. While the CART procedure is reimbursed in Japan given these advances and proven safety [4], global adoption remains limited. This is likely due to its uncertain clinical efficacy and higher procedural costs (737 vs. 19 USD), hindering adoption in other healthcare systems. The effectiveness of CART has been suggested by single‐arm observational studies [4], single‐institution reports [6], and a small feasibility trial involving only 20 patients, which lacked statistical power for efficacy comparison [13]. Thus, no large‐scale study has directly compared CART with paracentesis regarding mortality, QOL indicators such as functional outcomes, unplanned readmission, and healthcare resource utilization in patients with metastatic cancer.

This study aimed to compare the clinical outcomes and healthcare costs of CART with those of simple paracentesis among patients with metastatic solid cancers across Japan. Our findings could provide evidence to guide decision‐making for ascites management strategies.

## 2 Methods

### 2.1 Study Design and Data Source

This retrospective cohort study compared the clinical outcomes and healthcare costs of CART with those of paracentesis for ascites drainage among patients with metastatic solid cancers. Data were collected from a clinical database comprising 140 National Hospital Organization (NHO) hospitals across Japan [14]. The NHO maintains two databases: an administrative claims database and a clinical information database [15]. The former contains comprehensive information on patient demographics, medical costs, diagnosis, procedures, and medications. The latter includes daily records of medical charts, vital signs, and laboratory test results.

This study was performed in accordance with the Declaration of Helsinki and received ethical approval from the Institutional Review Board of Showa University (2023‐129‐A). Individual informed consent was not required owing to the retrospective design, with an opt‐out opportunity provided via the hospital website. The results were reported in adherence to the Strengthening the Reporting of Observational Studies in Epidemiology guidelines.

### 2.2 Participants

This study comprised adult patients (aged ≥ 18 years) with metastatic solid cancer admitted to NHO hospitals from April 2016 to March 2023. We included patients who were diagnosed with ascites at admission and subsequently underwent drainage with paracentesis or CART during hospitalization. Ascites was determined by the International Classification of Disease 10th Revision coding system (ICD‐10) in table S1 [16].

We excluded patients who underwent surgery under general anesthesia, were diagnosed with coronavirus disease 2019 (COVID‐19), had spontaneous bacterial peritonitis (SBP), had human immunodeficiency virus (HIV) infection, were pregnant, or had missing Barthel Index (BI) data (a standard measure of activities of daily living) at discharge. These exclusion criteria were applied because surgically treated patients might receive ascites drainage for postoperative chylous ascites [17]; patients with COVID‐19, SBP [6], HIV infection, or pregnancy may require non‐standard ascites drainage; and patients with missing BI data could not be evaluated for an outcome. ICD‐10 was used to classify COVID‐19, SBP [18], and HIV infection in Table S1.

Patients were classified into the CART or paracentesis groups based on the initial drainage procedure they received.

### 2.3 Outcome Variables

The primary outcomes were defined as a composite of three endpoints: in‐hospital mortality, functional disability at discharge, and 30‐day unplanned readmission [19]. We assessed functional disability only among survivors by classifying them as bedridden (discharge BI ≤ 35) [20] or severe dependence (discharge BI 40–60) [20, 21], on the condition that their discharge BI did not exceed their admission BI. BI evaluates the physical dependence on a 100‐point scale, with lower scores indicating more severe disability [21].

Secondary outcomes included total length of stay (LOS), rate of re‐intervention (albumin administration or re‐drainage within 2 weeks) [6], and healthcare costs. Costs were categorized as total, medical consultation, medication, surgical procedure, laboratory tests, hospital stay, and others, and were converted from Japanese yen to USD at a 150:1 exchange rate. Costs were calculated based on the medical fee at the time of treatment. The procedural costs included the technical fees and medical‐material fees.

### 2.4 Other Variables

To adjust for potential confounders, we collected data on the following covariates before the first ascites drainage. Patient characteristics comprised age [20, 22], sex [23], body mass index, and functional status [20] (bedridden [BI ≤ 35], severe dependence [BI 40–60], and mild or no dependence [BI ≥ 60]). Admission details encompassed primary cancer site [2] (colorectal, liver, pancreas, stomach, other digestive system, female genital organs, and others), admission after January 2020 (after the COVID‐19 pandemic), non‐home admission, and unplanned admission. Comorbidities and medical history included the Charlson Comorbidity Index (CCI) [16], comorbidities [22] (cerebrovascular disease, chronic pulmonary disease, congestive heart failure, diabetes, liver disease, and renal disease), medical history within 4 weeks prior to ascites drainage [22] (chemotherapy, radiation, surgery, diuretics, and opioids), medical history within 2 weeks prior to ascites drainage (albumin administration and outpatient drainage), and days from admission to first ascites drainage. Clinical status and laboratory values consisted of bacterial culture [13], palliative care intervention, oxygen inhalation, acetaminophen or non‐steroidal anti‐inflammatory drugs, antibiotics [13], steroids, body temperature, systolic blood pressure, and laboratory test values [2, 23] (serum albumin [Alb], absolute neutrophil count, creatinine, hemoglobin, potassium, sodium, total bilirubin, and white blood cell count). Specific cut‐off values for both vital signs and laboratory test values are presented in Table 1. Hospital characteristics [24] included the number of beds (< 300, 300–499, or ≥ 500 beds).

**TABLE 1:** Characteristics of patients with metastatic cancer before and after overlap weighting according to ascites drainage.

Variable	Before weighting			After weighting		
	Paracentesis	CART	SMD	Paracentesis	CART	SMD
	*n* = 702	*n* = 457		*n* = 702	*n* = 457	
Age, years, mean (SD)	68.8 (12.0)	67.0 (11.0)	0.15	67.5 (12.2)	67.5 (10.8)	< 0.01
Sex, *n* (%)			0.08			< 0.01
Male	376 (53.6)	227 (49.7)		355 (50.6)	231 (50.6)	
Female	326 (46.4)	230 (50.3)		347 (49.4)	226 (49.4)	
BMI, kg/m^2^, *n* (%)			0.10			< 0.01
< 18.5	110 (15.7)	76 (16.6)		113 (16.1)	74 (16.1)	
18.5–24.9	439 (62.5)	299 (65.4)		447 (63.7)	291 (63.7)	
≥ 25.0	153 (21.8)	82 (17.9)		142 (20.2)	92 (20.2)	
Functional status, *n* (%)			0.20			< 0.01
Mild or no dependence	526 (74.9)	374 (81.8)		555 (79.1)	362 (79.1)	
Severe dependence	94 (13.4)	54 (11.8)		89 (12.6)	58 (12.6)	
Bedridden	82 (11.7)	29 (6.3)		58 (8.2)	38 (8.2)	
Primary cancer site, *n* (%)			0.27			< 0.01
Colorectal	100 (14.2)	64 (14.0)		104 (14.8)	68 (14.8)	
Liver	60 (8.5)	17 (3.7)		32 (4.6)	21 (4.6)	
Pancreas	187 (26.6)	105 (23.0)		172 (24.6)	112 (24.6)	
Stomach	156 (22.2)	140 (30.6)		194 (27.7)	127 (27.7)	
Other digestive system	36 (5.1)	25 (5.5)		39 (5.6)	25 (5.6)	
Female genital organs	64 (9.1)	47 (10.3)		71 (10.1)	46 (10.1)	
Others	99 (14.1)	59 (12.9)		89 (12.7)	58 (12.7)	
After COVID‐19 pandemic, *n* (%)	290 (41.3)	206 (45.1)	0.08	302 (43.0)	197 (43.0)	< 0.01
Non‐home admission, *n* (%)	37 (5.3)	21 (4.6)	0.03	33 (4.7)	22 (4.7)	< 0.01
Unplanned admission, *n* (%)	409 (58.3)	239 (52.3)	0.12	385 (54.9)	251 (54.9)	< 0.01
CCI, *n* (%)			0.06			< 0.01
0	343 (48.9)	236 (51.6)		355 (50.6)	231 (50.6)	
≥ 1	359 (51.1)	221 (48.4)		347 (49.4)	226 (49.4)	
Cerebrovascular disease	24 (3.4)	10 (2.2)	0.07	16 (2.3)	11 (2.3)	< 0.01
Chronic pulmonary disease	57 (8.1)	27 (5.9)	0.09	43 (6.2)	28 (6.2)	< 0.01
Congestive heart failure	56 (8.0)	34 (7.4)	0.02	55 (7.8)	36 (7.8)	< 0.01
Diabetes	104 (14.8)	67 (14.7)	< 0.01	104 (14.8)	68 (14.8)	< 0.01
Liver disease	127 (18.1)	77 (16.8)	0.03	124 (17.7)	81 (17.7)	< 0.01
Renal disease	15 (2.1)	8 (1.8)	0.03	14 (2.0)	9 (2.0)	< 0.01
Medical history within 4 weeks^a^, *n* (%)						
Chemotherapy	282 (40.2)	209 (45.7)	0.11	321 (45.6)	209 (45.6)	< 0.01
Radiation	16 (2.3)	3 (0.7)	0.14	6 (0.8)	4 (0.8)	< 0.01
Surgery	70 (10.0)	46 (10.1)	< 0.01	70 (10.0)	46 (10.0)	< 0.01
Diuretic	383 (54.6)	265 (58.0)	0.07	394 (56.1)	256 (56.1)	< 0.01
Opioid	307 (43.7)	183 (40.0)	0.07	299 (42.6)	195 (42.6)	< 0.01
Medical history within 2 weeks^b^, *n* (%)						
Albumin preparation	63 (9.0)	36 (7.9)	0.04	61 (8.6)	39 (8.6)	< 0.01
Outpatient drainage	66 (9.4)	38 (8.3)	0.04	63 (9.0)	41 (9.0)	< 0.01
Bacterial culture, *n* (%)	265 (37.7)	137 (30.0)	0.16	229 (32.7)	149 (32.7)	< 0.01
Palliative care intervention, *n* (%)	64 (9.1)	31 (6.8)	0.09	53 (7.5)	34 (7.5)	< 0.01
Oxygen inhalation, *n* (%)	260 (37.0)	137 (30.0)	0.15	229 (32.7)	149 (32.7)	< 0.01
Acetaminophen or NSAIDs, *n* (%)	374 (53.3)	269 (58.9)	0.11	394 (56.2)	257 (56.2)	< 0.01
Antibiotics, *n* (%)	147 (20.9)	96 (21.0)	< 0.01	146 (20.8)	95 (20.8)	< 0.01
Steroid, *n* (%)	131 (18.7)	182 (39.8)	0.48	206 (29.3)	134 (29.3)	< 0.01
BT > 38°C, *n* (%)	75 (10.7)	93 (20.4)	0.27	105 (15.0)	69 (15.0)	< 0.01
sBP ≤ 100 mmHg, *n* (%)	312 (44.4)	240 (52.5)	0.16	350 (49.9)	228 (49.9)	< 0.01
Alb, g/dL, mean (SD)	2.5 (0.6)	2.5 (0.6)	0.03	2.5 (0.6)	2.5 (0.6)	< 0.01
Creatinine, mg/dL, mean (SD)	1.1 (0.8)	1.0 (0.7)	0.13	1.0 (0.7)	1.0 (0.7)	< 0.01
Hemoglobin, g/dL, mean (SD)	10.7 (2.5)	10.6 (2.5)	0.06	10.6 (2.4)	10.6 (2.5)	< 0.01
Potassium, mEq/mL, mean (SD)	4.3 (0.7)	4.3 (0.7)	0.10	4.3 (0.6)	4.3 (0.7)	< 0.01
Sodium, mEq/mL, mean (SD)	135.9 (5.1)	136.5 (4.8)	0.13	136.2 (5.0)	136.2 (4.7)	< 0.01
T‐Bil, mg/dL, mean (SD)	1.7 (3.2)	1.6 (2.7)	0.06	1.6 (2.9)	1.6 (2.8)	< 0.01
WBC > 12,000 cells/μL, *n* (%)	154 (21.9)	88 (19.3)	0.07	150 (21.3)	98 (21.3)	< 0.01
ANC < 1000 cells/μL, *n* (%)	30 (4.3)	33 (7.2)	0.13	38 (5.4)	25 (5.4)	< 0.01
Days until ascites drainage^c^, mean (SD)	7.5 (13.0)	5.4 (12.6)	0.17	6.3 (9.9)	6.3 (16.3)	< 0.01
Number of hospital beds, *n* (%)			0.24			< 0.01
< 300	71 (10.1)	26 (5.7)		51 (7.3)	33 (7.3)	
300–499	411 (58.5)	242 (53.0)		403 (57.4)	263 (57.4)	
≥ 500	220 (31.3)	189 (41.4)		248 (35.3)	161 (35.3)	

Abbreviations: Alb, serum albumin; ANC, absolute neutrophil count; BMI, body mass index; BT, body temperature; CART, cell‐free and concentrated ascites reinfusion therapy; CCI, Charlson Comorbidity Index; COVID‐19, coronavirus disease 2019; NSAIDs, non‐steroidal anti‐inflammatory drugs; sBP, systolic blood pressure; SD, standard deviation; SMD, standardized mean difference; T‐Bil, total bilirubin; WBC, white blood cell count.

^a^ “Medical history within 4 weeks” means that the patients underwent treatment within 4 weeks prior to ascites drainage after admission.

^b^ “Medical history within 2 weeks” means that the patients underwent treatment within 2 weeks prior to ascites drainage after admission.

^c^ “Days until ascites drainage” means the duration from admission to the first ascites drainage after admission.

Patient characteristics, admission details, CCI, comorbidities, and hospital characteristics were assessed at admission. Clinical status and laboratory values were assessed within 3 days before ascites drainage [25]. ICD‐10 [16] was used to classify the primary cancer site, CCI, and comorbidities (Table S1).

### 2.5 Statistical Analysis

To balance the baseline covariates between the CART and paracentesis groups, we used overlap propensity‐score weighting, which focuses on the population with clinical equipoise [26]. Propensity scores were estimated from a logistic regression model that included all variables as covariates. The balance of covariates before and after weighting was confirmed using the standardized mean difference (SMD), with an SMD value ≤ 0.1 indicating a good balance [27]. Missing values were imputed using a random forest‐based algorithm (“missRanger”) [28].

After weighting, clinical outcomes were compared between the groups. Continuous variables were summarized as mean and standard deviation or median and interquartile range (IQR) and were compared using the Wilcoxon rank‐sum test or the Welch test. Categorical variables were reported as proportions and were compared using the chi‐square test.

We used modified Poisson regression to estimate the risk ratio (RR) with 95% confidence intervals (95% CI) for in‐hospital mortality [29]. The composite outcomes of in‐hospital mortality, functional disability, and 30‐day unplanned readmission were assessed using a win‐ratio approach [30, 31]. This non‐parametric method was used to perform a generalized pairwise comparison between patients in the CART and paracentesis groups, where a ratio > 1 indicates a favorable result. In this analysis, these four outcomes were prioritized in the following order: (1) in‐hospital mortality, (2) bedridden, (3) severe dependence, and (4) 30‐day unplanned readmission (Figure S1). A win was recorded for the CART group if a patient in that group had a more favorable outcome. Given the possibility of many ties, win odds were also calculated.

To evaluate for potential heterogeneity, we performed subgroup analyses stratified by age (18–64 or ≥ 65 years) [22], sex [23], Alb (≤ 2.5 or > 2.5 g/dL) [32], chemotherapy (yes or no) [33], and primary cancer site (gastrointestinal or non‐gastrointestinal) [34]. Moreover, we assessed the robustness of our findings through the following sensitivity analyses: (1) a complete case analysis to assess for potential bias introduced by our missing‐data imputation strategy; (2) an analysis restricted to patients receiving their first drainage within 3 days after admission to account for early treatment effect [25]; (3) an analysis limited to patients with LOS ≤ 28 days to mitigate the effect of prolonged stays; (4) an analysis including patients with missing BI data (for the mortality outcome only); and (5) an analysis with a modified Poisson regression using a mixed‐effects model clustered by hospital to account for facility‐level heterogeneity. Additionally, to assess the potential impact of unmeasured confounders on in‐hospital mortality, we calculated the E‐value [35].

All statistical analyses were performed using R version 4.3.1 (R Foundation for Statistical Computing, Vienna, Austria). A two‐sided *p* < 0.05 was considered statistically significant.

## 3 Results

**FIGURE 2:**
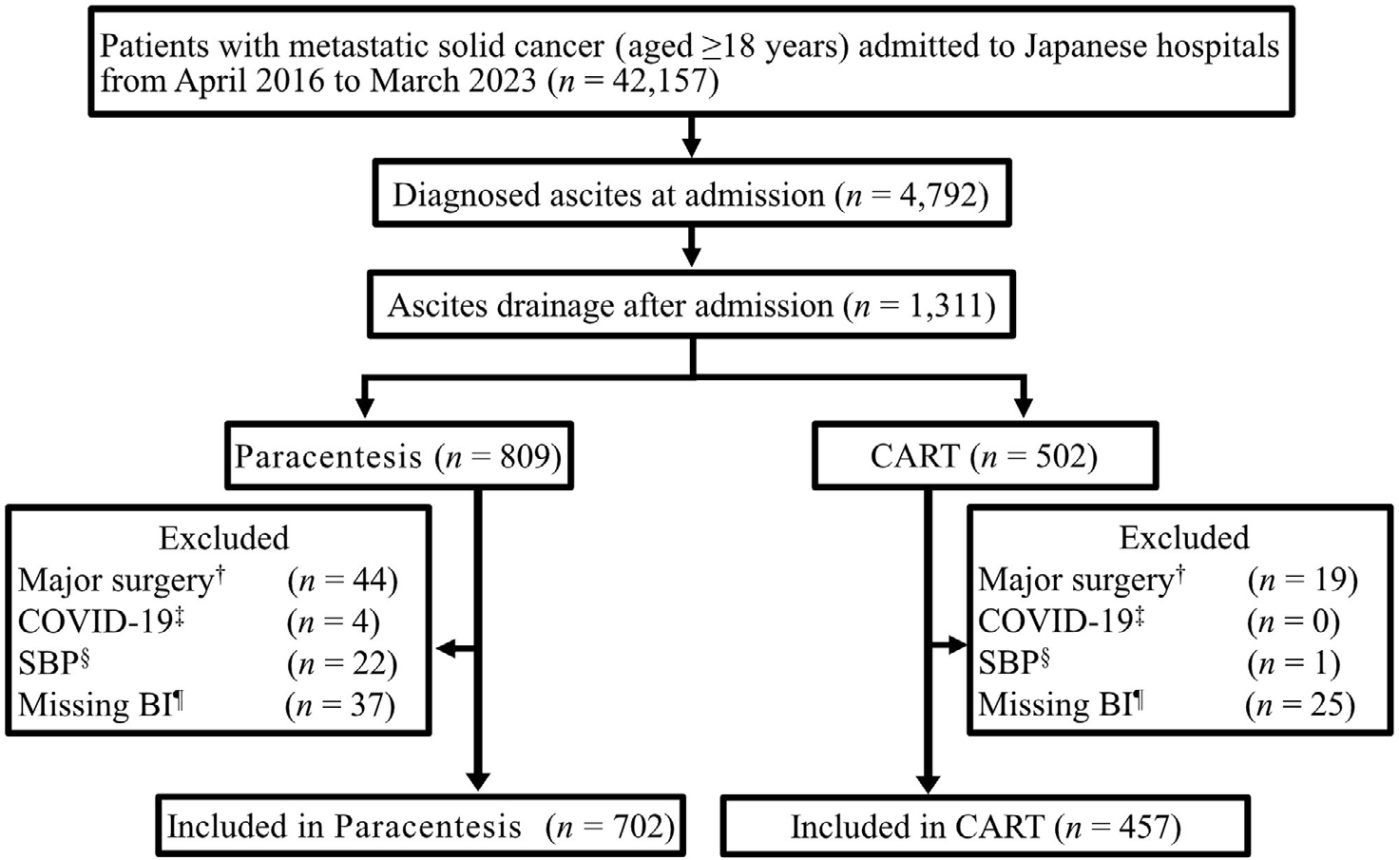
Flow diagram of the enrollment of study participants. ^†^“Major surgery” means that the patients underwent surgery under general anesthesia during hospitalization. ^‡^“COVID‐19” means that the patients were diagnosed with COVID‐19 during hospitalization. ^§^“SBP” means that the patients were diagnosed with spontaneous bacterial peritonitis during hospitalization. ^¶^“Missing BI” means patients with missing Barthel Index data at discharge. CART, cell‐free and concentrated ascites reinfusion therapy; COVID‐19, coronavirus disease 2019.

Of the 42,157 patients, 1311 met the initial eligibility criteria. Of them, 152 patients (11.6%) were excluded and 1159 patients (paracentesis: 702, CART: 457) were included from 51 hospitals, with no cases of pregnancy or HIV infection in either group (Figure 2). We imputed 12 missing values with a maximum missing rate of < 30% (Table S2). All baseline characteristics were well balanced between the groups after weighting, as indicated by SMD < 0.01 for all variables (Table 1).

**FIGURE 3:**
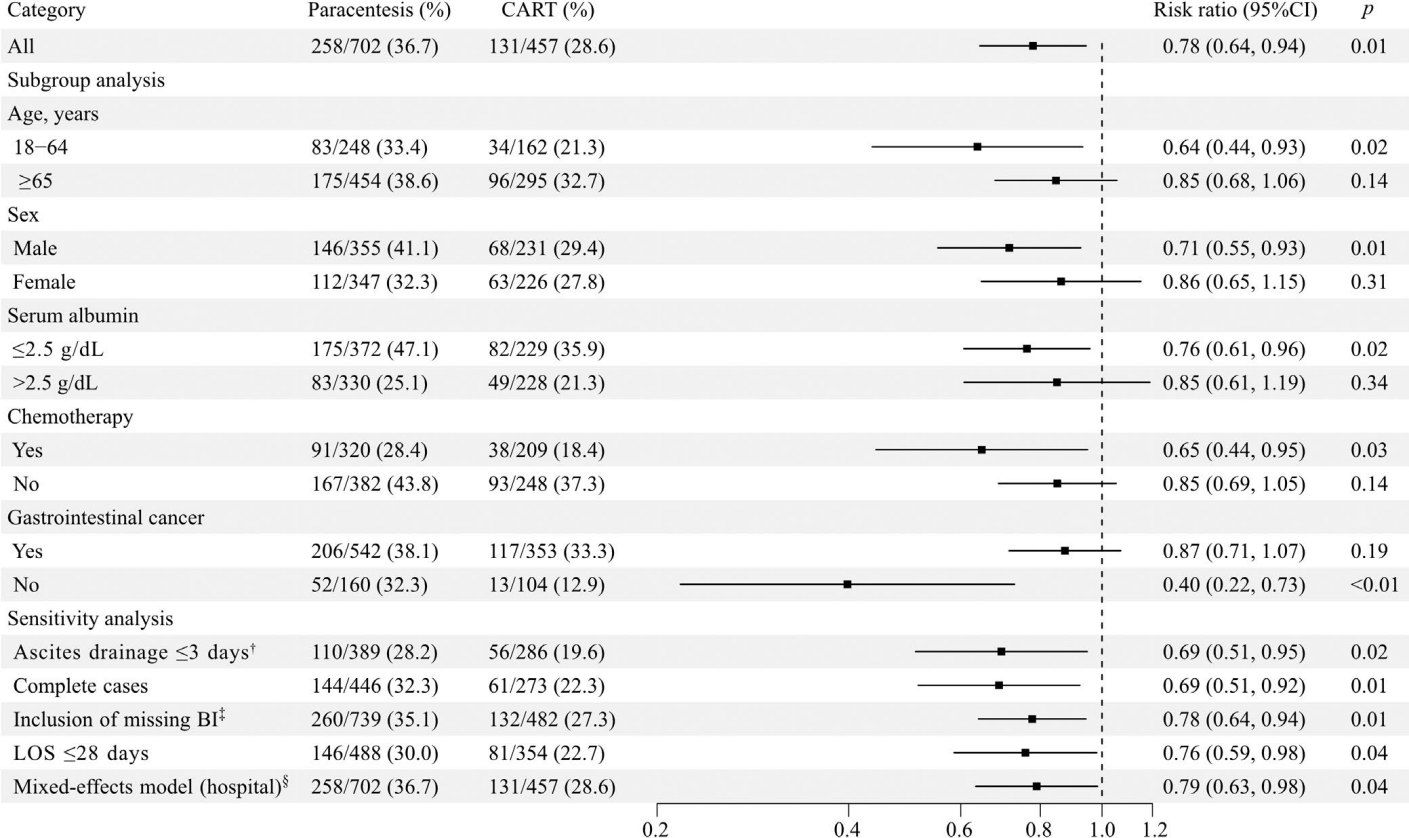
Forest plot showing the risk ratios by subgroup and sensitivity analyses after overlap weighting. ^†^“Ascites drainage ≤ 3 days” means that the patients were started on ascites drainage within 3 days after admission. ^‡^“Inclusion of missing BI” means that we added patients who had missing Barthel Index data at discharge. ^§^“Mixed‐effects model (hospital)” means that we conducted a modified Poisson regression using a mixed‐effects model clustered by hospital. 95% CI, 95% confidence interval; Alb, serum albumin; CART, cell‐free and concentrated ascites reinfusion therapy; LOS, length of stay from admission to discharge.

After weighting, the in‐hospital mortality rates were 36.7% (258/702) and 28.6% (131/457) in the paracentesis and CART groups, respectively (absolute risk difference: 8.1%). The CART group had a lower mortality rate than the paracentesis group (RR: 0.78, 95% CI: 0.64–0.94, *p* < 0.01). In several subgroups, CART was associated with lower in‐hospital mortality in patients aged 18–64 years (RR: 0.64, 95% CI: 0.44–0.93, *p* = 0.02), males (RR: 0.71, 95% CI: 0.55–0.93, *p* = 0.01), those with Alb ≤ 2.5 g/dL (RR: 0.76, 95% CI: 0.61–0.96, *p* = 0.02), those who received chemotherapy (RR: 0.65, 95% CI: 0.44–0.95, *p* = 0.03), and those with non‐gastrointestinal cancer (RR: 0.40, 95% CI: 0.22–0.73, *p* < 0.01). However, no significant difference was observed among other subgroups (Figure 3). The results of sensitivity analysis were consistent with those of the overall analysis. Additionally, the *E*‐value for the overall analysis was 1.89 (95% CI: 1.31–2.49).

**FIGURE 4:**
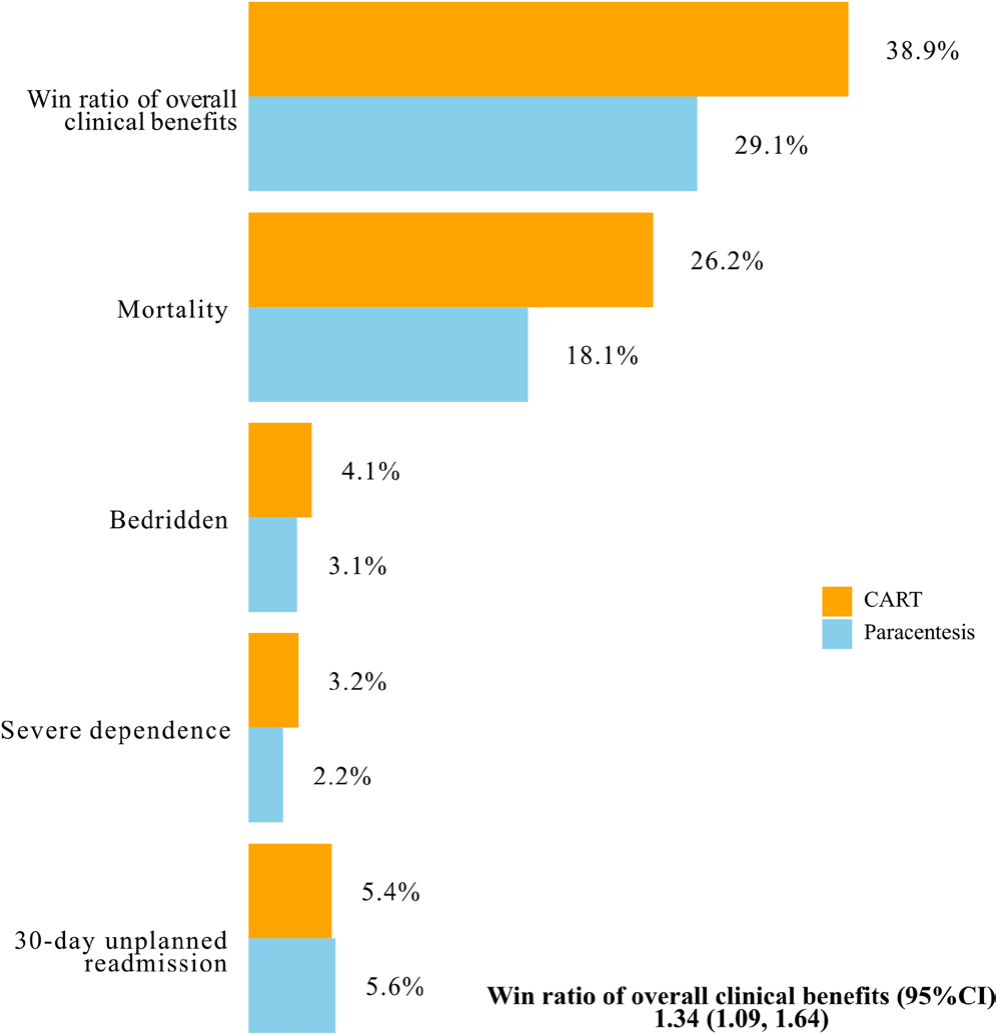
Adjusted win ratio of the composite primary outcome and its components using a non‐parametric generalized pairwise comparison after overlap weighting. 95% CI, 95% confidence interval; CART, cell‐free and concentrated ascites reinfusion therapy.

The win‐ratio analysis for the composite primary outcomes showed that the CART group had more favorable outcomes than the paracentesis group (38.9% vs. 29.1%), with a win ratio of 1.34 (95% CI: 1.09–1.64, *p* < 0.01). The CART group showed higher win rates for mortality (26.2% vs. 18.1%), bedridden status (4.1% vs. 3.1%), and severe dependence (3.2% vs. 2.2%) than the paracentesis group. The win rates for 30‐day unplanned readmission were similar (5.4% vs. 5.6%) between the CART and paracentesis groups (Figure 4).

In subgroup analyses, the CART group showed a higher win ratio than the paracentesis group in patients aged ≥ 65 years (1.30, 95% CI: 1.01–1.67, *p* = 0.04), males (1.48, 95% CI: 1.11–1.97, *p* < 0.01), those with Alb ≤ 2.5 g/dL (1.51, 95% CI: 1.13–2.02, *p* < 0.01), those who received chemotherapy (1.37, 95% CI: 1.00–1.88, *p* = 0.05), and those with non‐gastrointestinal cancer (1.87, 95% CI: 1.22–2.86, *p* < 0.01). Conversely, the benefit of the CART group was not significant in the other subgroups. Although the higher win rates for mortality in the CART group were consistent across all subgroups, the win rates for the other primary outcomes varied depending on the subgroup (Table S3). The sensitivity analyses showed results consistent with those of the overall analysis (Figure S2A). The win‐odds analysis showed similar patterns to the win‐ratio analysis (Figure S2B).

The median LOS was shorter in the CART group than in the paracentesis group (14.1 days [IQR: 5.0–27.0] vs. 19.0 days [IQR: 11.0–32.0], *p* < 0.01). The CART group also had lower re‐intervention rates for both albumin administration (11.6% vs. 17.3%, *p* = 0.01) and re‐drainage (32.7% vs. 52.7%, *p* < 0.01). The median total cost was lower in the CART group (4490.9 USD [IQR: 2042.3–7054.5] vs. 5084.1 USD [IQR: 3054.7–8659.7], *p* < 0.01). However, the cost of the surgical procedure was significantly higher in the CART group (Table 2).

**TABLE 2:** Comparison of secondary outcomes between the paracentesis and CART groups after overlap weighting.

Secondary outcomes	Paracentesis	CART	*p*
	*n* = 702	*n* = 457	
LOS, median [IQR]	19.0 [10.0, 32.0]	14.1 [5.0, 27.0]	< 0.01
Re‐intervention within 2 weeks after first ascites drainage, *n* (%)			
Albumin administration	121 (17.3)	53 (11.6)	0.02
Re‐drainage	370 (52.7)	150 (32.7)	< 0.01
Cost, USD, median [IQR]			
Total	5084.1 [3054.7, 8659.7]	4490.9 [2042.3, 7054.5]	< 0.01
Medical consultation	72.1 [38.3, 127.1]	58.4 [26.7, 118.6]	< 0.01
Medication	515.1 [177.0, 1557.3]	291.0 [48.5, 935.5]	< 0.01
Surgical procedure	122.4 [34.1, 816.6]	799.3 [762.2, 1346.7]	< 0.01
Laboratory tests	420.0 [216.7, 832.8]	302.2 [122.8, 631.3]	< 0.01
Hospital stay	3128.3 [1762.6, 4976.3]	2270.9 [894.8, 4123.8]	< 0.01
Others	0.0 [0.0, 13.7]	0.0 [0.0, 0.0]	0.14

Abbreviations: CART, cell‐free and concentrated ascites reinfusion therapy; IQR, interquartile range; LOS, length of stay from admission to discharge.

## 4 Discussion

In this study, CART was associated with lower in‐hospital mortality and better composite outcomes compared with paracentesis in hospitalized patients with metastatic solid cancer, particularly in males, patients with Alb ≤ 2.5 g/dL, and those with non‐gastrointestinal cancer. The robustness of these findings was supported by sensitivity analyses. Additionally, CART was associated with shorter LOS, reduced albumin consumption, prolonged time to re‐drainage, and lower total costs, despite its higher procedural cost.

This study provides the first large‐scale evidence that CART is associated with lower in‐hospital mortality compared with paracentesis. While previous research has primarily focused on improvements in performance status, QOL‐related symptoms, serum albumin levels, or the period until re‐drainage [4, 6, 13, 36, 37], the impact of CART on hard clinical endpoints—including survival, LOS, and total costs—has remained unverified. The survival benefit may be attributed to the ability of CART to prevent severe hypoalbuminemia, which enables safe, large‐volume drainage [4], potentially creating opportunities for subsequent chemotherapy [12, 38].

Previous single‐arm or small‐scale studies have reported improvements in performance status and symptom control among surviving patients [6, 36, 37]. However, our study is the first to show that overall outcomes, including functional status and 30‐day unplanned admission, significantly favored CART in a large‐scale analysis. An examination of these components suggests that the CART group tended to have a better functional status, which is consistent with prior findings of symptom relief and improved performance status [6, 36, 37]. In contrast, the win rates for 30‐day unplanned readmission were comparable, highlighting the temporary effect of both drainage strategies [6]. Additionally, our subgroup analyses provide insight into which patients might derive the most benefit from CART. A pronounced advantage of CART in both mortality and composite outcomes was observed among males and patients with low serum albumin levels, which are previously identified predictors of a poorer prognosis [2, 23]. Males generally have greater skeletal muscle mass and higher basal metabolic rates than females, making them more susceptible to severe nutritional depletion from paracentesis. CART mitigates this nutritional depletion, potentially explaining the greater survival benefit in this population. Similarly, this mechanism may extend to patients with hypoalbuminemia because their limited nutritional reserves make them vulnerable to further protein loss. Furthermore, regarding cancer types, our subgroup analysis showed the benefit of CART in patients with non‐gastrointestinal cancer. This finding is consistent with a recent study suggesting that CART is more effective for patients with gynecological cancer than non‐gynecological cancer (mainly gastrointestinal cancer) [34]. Thus, prioritizing CART for patients with high‐risk or specific cancer types may be a reasonable strategy in resource‐limited settings.

The secondary outcomes demonstrated that CART may improve both clinical and healthcare cost efficacy. Regarding clinical efficacy, the reduction in re‐interventions (albumin and re‐drainage) is consistent with prior studies [6] and indicates improved symptom control and reduced patient burden. In terms of healthcare cost efficiency, despite the higher procedural costs of CART, it was associated with a shorter LOS and lower total in‐hospital costs. This suggests that the higher initial investment for CART is offset by the reduction in subsequent resource utilization, such as hospital stays and albumin consumption. Although outpatient paracentesis is less expensive for manageable ascites [39], our findings indicate that CART may be a superior option for patients with severe ascites requiring admission.

This study had some limitations. First, our analysis was based on Japanese hospital data under the universal health insurance system, which may limit the generalizability of our findings to other countries with different healthcare and reimbursement systems. However, given that CART is not widely disseminated outside of Japan [6], large‐scale international research is currently not feasible. Consequently, our large‐scale, real‐world study may represent the only available evidence for healthcare systems considering the adoption of CART.

Second, although the propensity score method was used to balance the 40 potential confounders, unmeasured confounders, including ascites volume, frailty, patient preference, hospital expertise, and physician instructions for life‐sustaining treatment, could have influenced our results [6, 38]. Given that the lower limit of the 95% CI for the *E*‐value was 1.31 (< 2.0) [35], these factors could have biased the results. For example, if CART is preferentially used in patients with a greater ascites burden, our findings may underestimate its true benefit. Conversely, if a physician chooses paracentesis for patients predicted to have only a short life expectancy considering the procedure cost for CART, the benefit of CART could be overestimated.

Third, our database lacked QOL scores or symptom burden measures. While we used the BI to assess functional disability, we could not evaluate symptoms such as abdominal distension or dyspnea. This limits the evaluation of patient benefit regarding symptom relief.

Fourth, our database did not contain procedure‐related adverse events, such as infection, bleeding, or electrolyte imbalance. Although previous studies have suggested the safety of CART [12, 13], we could not compare the incidence of these complications between the groups in this study. Future prospective studies are needed to evaluate the safety profile.

Finally, our assessment of post‐discharge outcomes was incomplete. Although our database could identify unplanned readmissions to the same hospital, it could not capture events that occurred after discharge, including out‐of‐hospital mortality, admissions to other facilities, or post‐discharge costs. This limitation may lead to an underestimation of mortality, 30‐day unplanned readmission rates, and post‐discharge costs. In the future, long‐term prospective studies are needed to assess post‐discharge outcomes and conduct a cost‐effectiveness analysis of CART versus paracentesis to compare the results against the willingness‐to‐pay thresholds [40] of each country, supporting international adoption.

## 5 Conclusions

This is the first large‐scale study to demonstrate that CART is associated with improvements in clinical outcomes and healthcare costs compared with paracentesis among hospitalized patients with metastatic solid cancer, particularly in males, patients with serum albumin ≤ 2.5 g/dL, and those with non‐gastrointestinal cancer. Our findings may support a strategic approach to managing malignant ascites by prioritizing CART for patients with poor prognostic factors, optimizing both clinical outcomes and resource use.

## Author Contributions

**Yuki Hashimoto:** conceptualization (equal), data curation (equal), formal analysis (equal), funding acquisition (equal), investigation (equal), methodology (equal), project administration (equal), resources (equal), software (equal), validation (equal), visualization (equal), writing – original draft (equal), writing – review and editing (equal). **Norihiko Inoue:** data curation (equal), methodology (equal), software (equal), supervision (equal). **Shinobu Imai:** conceptualization (equal), supervision (equal), writing – review and editing (equal).

## Funding

This study was partially funded by JSPS KAKENHI (Grants 23K19882 and 24K20157).

## Ethics Statement

This study was performed in accordance with the Declaration of Helsinki and received ethical approval from the Institutional Review Board of Showa University (2023‐129‐A). Individual informed consent was not required owing to the retrospective design, with an opt‐out opportunity provided via the hospital website.

## Conflicts of Interest

The authors declare no conflicts of interest.

## Supporting information


**Data S1:** cam471491‐sup‐0001‐supinfo.docx.

## Data Availability

The datasets generated or analyzed during this study are not publicly available because they contain sensitive personal information. The analytical code is available from the corresponding author upon request.
