# Supplementary material for: Clinical Outcomes and Healthcare Costs of CART Versus Paracentesis for Malignant Ascites: A Nationwide Retrospective Cohort Study in Japan
Source: Cancer Med. 2025 Dec 26;15(1):e71491. doi: 10.1002/cam4.71491 (PMC12743171; doi:10.1002/cam4.71491)
Supplement: Supplementary file 1 — Data S1: cam471491‐sup‐0001‐supinfo.docx. [file CAM4-15-e71491-s001.docx]

Table S1. ICD-10 codes of the variables used as covariates

| Disease | ICD-10 codes |
| --- | --- |
| Participant criteria |  |
| Ascites | C786, R18 |
| Coronavirus disease 2019 | U071 |
| HIV | B20, B21, B22, B24 |
| SBP | K65 |
| Primary cancer site |  |
| Colorectal | C18, C19, C20, C785 |
| Liver | C22, C787 |
| Pancreas | C25 |
| Stomach | C16 |
| Other digestive system | C15, C17, C21, C23, C24, C26 |
| Female genital organs | C51, C52, C53, C54, C55, C56, C57, C58 |
| Others | C00, C01, C02, C03, C04, C05, C06, C07, C08, C09, C10, C11, C12, C13, C14, C30, C31, C32, C33, C34, C37, C38, C39, C40, C41, C43, C44, C45, C46, C47, C48, C49, C50, C60, C61, C62, C63, C64, C65, C66, C67, C68, C69, C70, C71, C72, C73, C74, C75, C76, C77, C780, C781, C782, C783, C784, C786, C788, C79, C80, C96, C97 |
| Comorbidities |  |
| Cerebrovascular disease | G45, G46, H340, I60, I61, I62, I63, I64, I65, I66, I67, I68, I69 |
| Chronic pulmonary disease | I278, I279, J40, J41, J42, J43, J44, J45, J46, J47, J60, J61, J62, J63, J64, J65, J66, J67, J684, J701, J703 |
| Congestive heart failure | I099, I110, I130, I132, I255, I420, I425, I426, I427, I428, I429, I43, I50, P290 |
| Diabetes | E100, E101, E102, E103, E104, E105, E106, E107, E108, E109, E110, E111, E112, E113, E114, E115, E116, E117, E118, E119, E120, E121, E122, E123, E124, E125, E126, E127, E128, E129, E130, E131, E132, E133, E134, E135, E136, E137, E138, E139, E140, E141, E142, E143, E144, E145, E146, E147, E148, E149 |
| Liver disease | B18, I850, I859, I864, I982, K700, K701, K702, K703, K704, K709, K711, K713, K714, K715, K717, K721, K729, K73, K74, K760, K762, K763, K764, K765, K766, K767, K768, K769, Z944 |
| Renal disease | I120, I131, N032, N033, N034, N035, N036, N037, N052, N053, N054, N055, N056, N057, N18, N19, N250, Z490, Z491, Z492, Z940, Z992 |

HIV = human immunodeficiency virus; ICD-10 = International Classification of Disease 10th Revision coding system; SBP = spontaneous bacterial peritonitis.

Table S2. Missing variables among patients with metastatic cancer

| Missing variable, n (%) | Paracentesis  *n* = 702 | CART  *n* = 457 | SMD |
| --- | --- | --- | --- |
| BMI | 14 (2.0) | 10 (2.2) | 0.01 |
| Functional status at admission | 62 (8.8) | 37 (8.1) | 0.03 |
| BT | 11 (1.6) | 1 (0.2) | 0.14 |
| sBP | 11 (1.6) | 1 (0.2) | 0.14 |
| Alb | 149 (21.2) | 112 (24.5) | 0.08 |
| ANC | 175 (24.9) | 130 (28.4) | 0.08 |
| Creatinine | 110 (15.7) | 93 (20.4) | 0.12 |
| Hemoglobin | 108 (15.4) | 84 (18.4) | 0.08 |
| Potassium | 111 (15.8) | 92 (20.1) | 0.11 |
| Sodium | 111 (15.8) | 92 (20.1) | 0.11 |
| T-Bil | 126 (17.9) | 100 (21.9) | 0.10 |
| WBC | 116 (16.5) | 89 (19.5) | 0.08 |

Alb = serum albumin; ANC = absolute neutrophil count; BMI = body mass index; BT = body temperature; CART = cell-free and concentrated ascites reinfusion therapy; sBP = systolic blood pressure; SMD = standardized mean difference; T-Bil = total bilirubin; WBC = white blood cell count.

Table S3. Win ratio of each primary outcome in patients who underwent paracentesis or CART for ascites drainage after overlap weighting

|  | Overall | | Mortality | | Bedridden | | Severe dependence | | 30-day unplanned readmission | |
| --- | --- | --- | --- | --- | --- | --- | --- | --- | --- | --- |
| Category | Paracentesis | CART | Paracentesis | CART | Paracentesis | CART | Paracentesis | CART | Paracentesis | CART |
| All (%) | 29.1 | 38.9 | 18.1 | 26.2 | 3.1 | 4.1 | 2.2 | 3.2 | 5.6 | 5.4 |
| Subgroup analysis (%) |  |  |  |  |  |  |  |  |  |  |
| Age, years |  |  |  |  |  |  |  |  |  |  |
| 18–64 | 28.2 | 39.3 | 14.2 | 26.3 | 2.3 | 4.4 | 4.0 | 2.9 | 7.7 | 5.6 |
| ≥65 | 29.6 | 38.4 | 20.1 | 26.0 | 3.5 | 3.9 | 1.4 | 3.3 | 4.6 | 5.2 |
| Sex |  |  |  |  |  |  |  |  |  |  |
| Male | 27.5 | 40.6 | 17.3 | 29.0 | 2.0 | 4.1 | 2.0 | 2.3 | 6.1 | 5.2 |
| Female | 30.7 | 37.0 | 18.9 | 23.3 | 4.4 | 4.1 | 2.5 | 4.1 | 5.0 | 5.5 |
| Alb |  |  |  |  |  |  |  |  |  |  |
| ≤2.5 g/dL | 27.2 | 41.2 | 19.0 | 30.2 | 3.8 | 5.3 | 1.1 | 2.8 | 3.4 | 3.0 |
| >2.5 g/dL | 30.2 | 33.9 | 16.0 | 19.7 | 1.6 | 1.9 | 3.9 | 3.6 | 8.7 | 8.6 |
| Chemotherapy |  |  |  |  |  |  |  |  |  |  |
| Yes | 27.7 | 38.1 | 13.1 | 23.1 | 2.4 | 3.3 | 3.1 | 3.9 | 9.0 | 7.6 |
| No | 29.3 | 38.2 | 20.9 | 27.5 | 3.4 | 4.4 | 1.6 | 2.6 | 3.3 | 3.8 |
| Gastrointestinal cancer |  |  |  |  |  |  |  |  |  |  |
| Yes | 30.8 | 37.5 | 20.6 | 25.4 | 2.7 | 4.1 | 1.8 | 3.0 | 5.6 | 5.0 |
| No | 22.7 | 42.4 | 8.7 | 28.1 | 4.5 | 3.7 | 4.0 | 4.1 | 5.5 | 6.5 |
| Sensitivity analysis (%) |  |  |  |  |  |  |  |  |  |  |
| Complete cases | 28.7 | 39.2 | 15.9 | 23.2 | 3.3 | 4.3 | 2.1 | 3.9 | 7.4 | 7.8 |
| Ascites drainage ≤3 days^†^ | 26.9 | 38.7 | 14.1 | 22.7 | 2.6 | 3.8 | 1.7 | 4.5 | 8.5 | 7.7 |
| LOS ≤28 days | 28.7 | 39.2 | 15.9 | 23.2 | 3.3 | 4.3 | 2.1 | 3.9 | 7.4 | 7.8 |

^†^ “Ascites drainage ≤3 days” means that the patients were started on ascites drainage within 3 days after admission.

Alb = serum albumin; CART = cell-free and concentrated ascites reinfusion therapy; LOS = length of stay from admission to discharge.


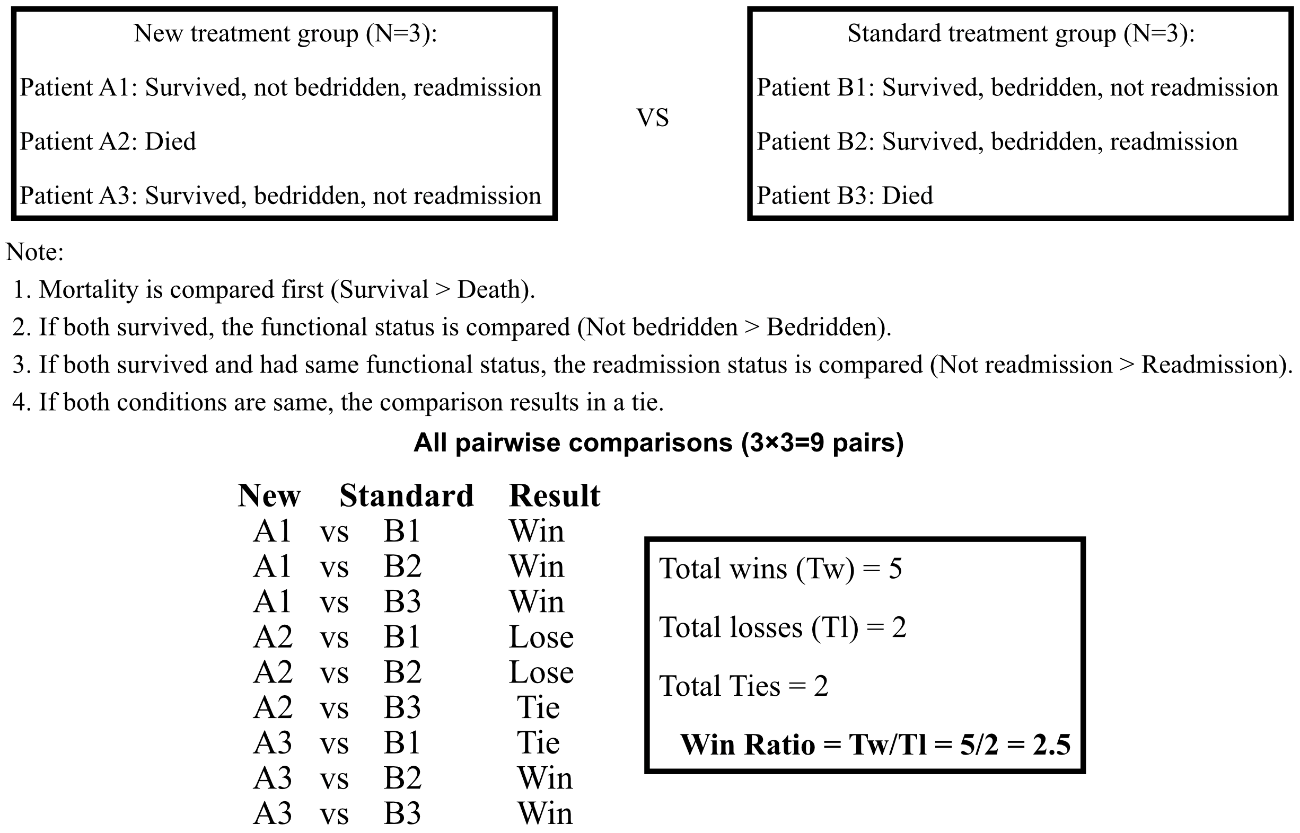


Figure S1. Schematic diagram of the win ratio approaches for the composite endpoints (death, functional status, and readmission).


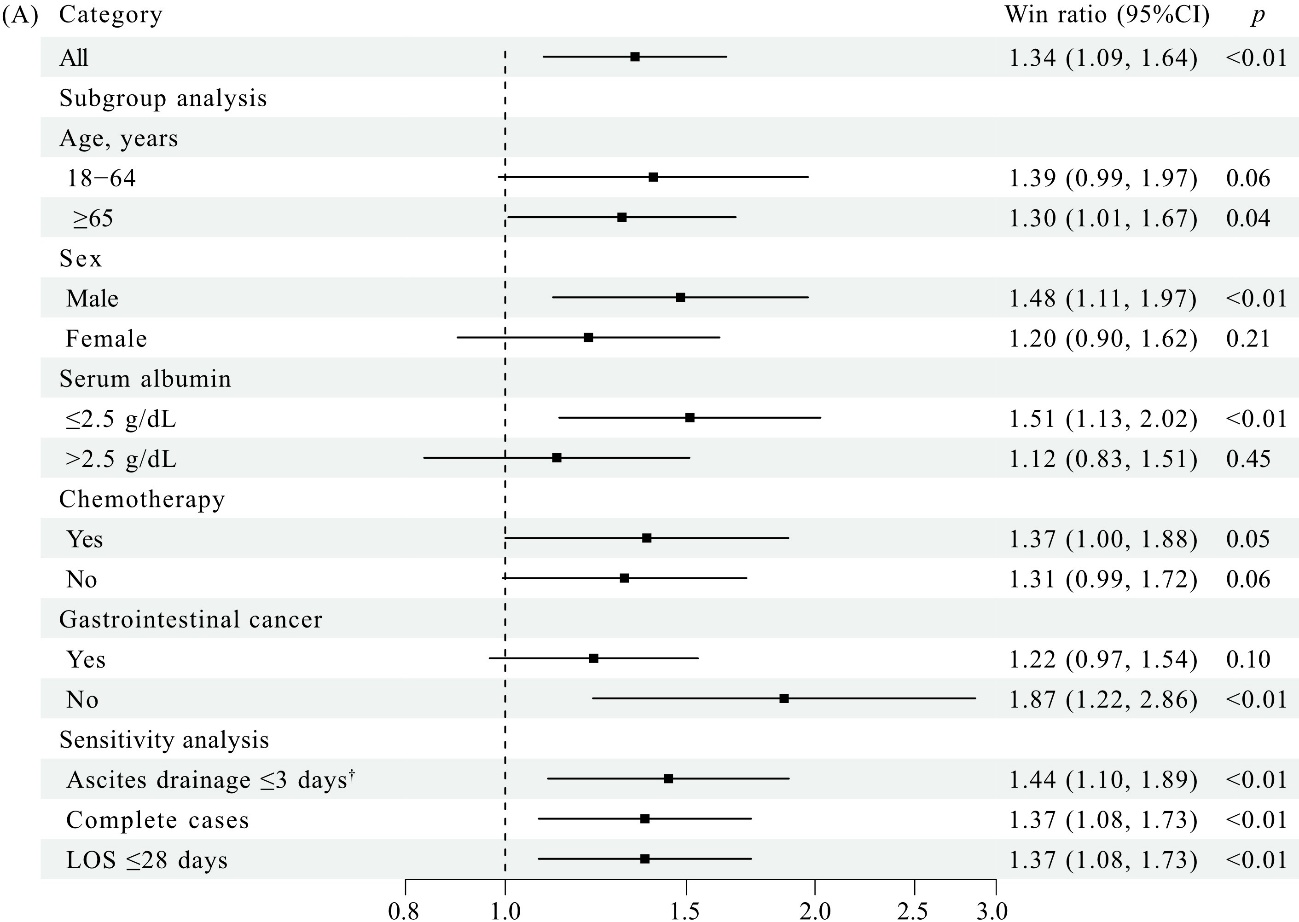


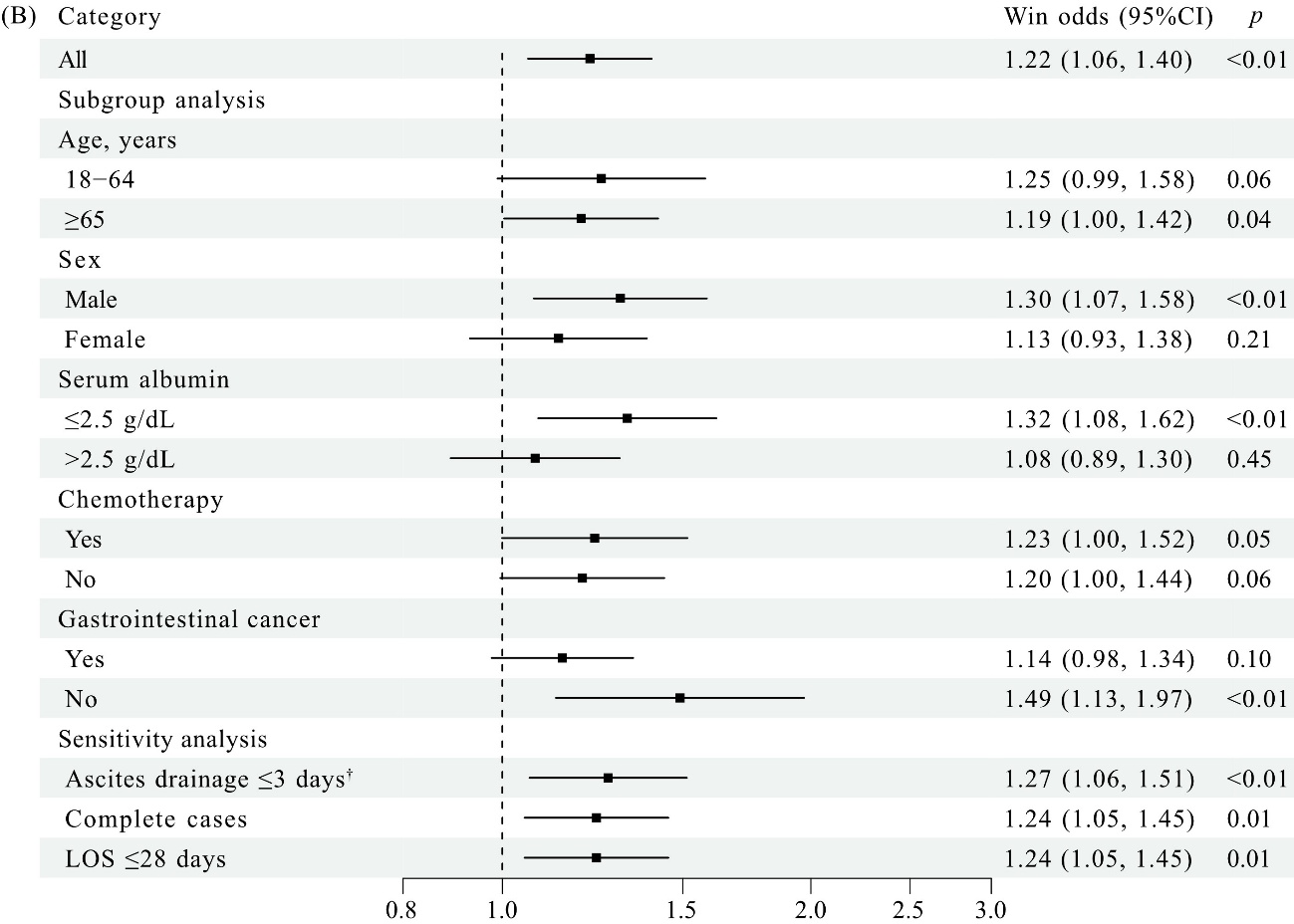


Figure S2. Forest plot showing the win-ratio approaches by subgroup and sensitivity analyses after overlap weighting.

(A) Forest plot showing the win ratios.

(B) Forest plot showing the win odds.

^†^ “Ascites drainage ≤3 days” means that the patients were started on ascites drainage within 3 days after admission.

Alb = serum albumin; CART = cell-free and concentrated ascites reinfusion therapy; LOS = length of stay from admission to discharge.
